# Combined Effects of Feedforward Inhibition and Excitation in Thalamocortical Circuit on the Transitions of Epileptic Seizures

**DOI:** 10.3389/fncom.2017.00059

**Published:** 2017-07-07

**Authors:** Denggui Fan, Lixia Duan, Qian Wang, Guoming Luan

**Affiliations:** ^1^School of Mathematics and Physics, University of Science and Technology BeijingBeijing, China; ^2^Beijing Key Laboratory for Magneto-Photoelectrical Composite and Interface Science, University of Science and Technology BeijingBeijing, China; ^3^Beijing Key Laboratory of Knowledge Engineering for Materials Science, University of Science and Technology BeijingBeijing, China; ^4^School of Science, North China University of TechnologyBeijing, China; ^5^Beijing Key Laboratory of Epilepsy, Epilepsy Center, Department of Functional Neurosurgery, Sanbo Brain Hospital, Capital Medical UniversityBeijing, China; ^6^Beijing Institute for Brain DisordersBeijing, China

**Keywords:** thalamocortical circuit, feedforward excitation and inhibition, epileptic seizures, tonic-clonic oscillations, spike and wave discharges, fast-slow dynamics

## Abstract

The mechanisms underlying electrophysiologically observed two-way transitions between absence and tonic-clonic epileptic seizures in cerebral cortex remain unknown. The interplay within thalamocortical network is believed to give rise to these epileptic multiple modes of activity and transitions between them. In particular, it is thought that in some areas of cortex there exists feedforward inhibition from specific relay nucleus of thalamus (TC) to inhibitory neuronal population (IN) which has even more stronger functions on cortical activities than the known feedforward excitation from TC to excitatory neuronal population (EX). Inspired by this, we proposed a modified computational model by introducing feedforward inhibitory connectivity within thalamocortical circuit, to systematically investigate the combined effects of feedforward inhibition and excitation on transitions of epileptic seizures. We first found that the feedforward excitation can induce the transition from tonic oscillation to spike and wave discharges (SWD) in cortex, i.e., the epileptic tonic-absence seizures, with the fixed weak feedforward inhibition. Thereinto, the phase of absence seizures corresponding to strong feedforward excitation can be further transformed into the clonic oscillations with the increasing of feedforward inhibition, representing the epileptic absence-clonic seizures. We also observed the other fascinating dynamical states, such as periodic 2/3/4-spike and wave discharges, reversed SWD and clonic oscillations, as well as saturated firings. More importantly, we can identify the stable parameter regions representing the tonic-clonic oscillations and SWD discharges of epileptic seizures on the 2-D plane composed of feedforward inhibition and excitation, where the physiologically plausible transition pathways between tonic-clonic and absence seizures can be figured out. These results indicate the functional role of feedforward pathways in controlling epileptic seizures and the modified thalamocortical model may provide a guide for future efforts to mechanistically link feedforward pathways in the pathogenesis of epileptic seizures.

## 1. Introduction

Epilepsy is a debilitating condition of chronic neurological unprovoked seizures (Kramer et al., 2005; Vaurio et al., 2017), which can induce the cognitive, linguistic and behavioral disorders (Barnes and Paolicchi, 2008; Caplan et al., 2008), extending well beyond the immediate effect from seizures (Vaurio et al., 2017). The seizure classifications of epilepsy can be divided into simple partial seizures (Devinsky et al., 1989; Kim et al., 2016), complex partial seizures (Jeniffer et al., 2015; Mohamed and Burnham, 2016) and generalized seizures (Youngblood et al., 2015). Experimental and computational evidences have suggested that these seizures are closely related to each other (Devinsky et al., 1988; Rogers et al., 2012; Varon Perez et al., 2012; Fan et al., 2015; Li et al., 2016a,b; Liu et al., 2016). This could be attributable to concomitance of multiple intricate mechanisms.

**Figure 1:**
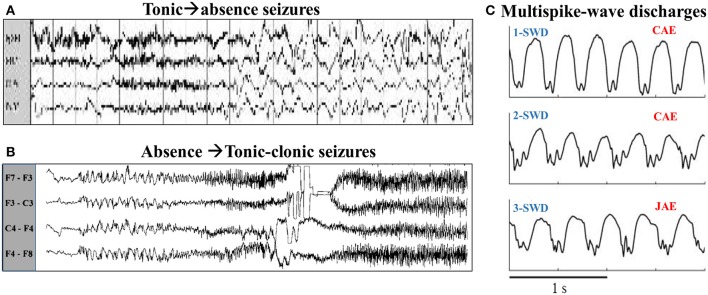
**(A)** The typical tonic-absence EEG pattern, modified from Shih and Hirsch (2003). **(B)** Electrographic evolution of an absence seizure to generalized tonic-clonic activity in patient, modified from Mayville et al. (2000). **(C)** EEG recordings from three different patients with an absence seizure, modified from Marten et al. (2009). The first two patients are diagnosed with CAE (childhood absence epilepsy) (Upper and Middle panels), while the patient in the lower panel is diagnosed with JAE (juvenile absence epilepsy).

Absence and tonic-clonic seizures are both the form of generalized epileptic seizures, i.e., the involvements of whole cortical region in humans during the pathological brain rhythms of epileptic seizures. In the electroencephalogram (EEG), generalized absence seizures of epilepsy, mainly occurring in children and juvenile (Marten et al., 2009), are typically characterized by bilaterally highly synchronized 2–4 Hz spike and wave discharges (SWD) (Meeren et al., 2002; Sitnikova, 2010) with a brief impairment of consciousness (i.e., absence) (Panayiotopoulos, 2001; Sitnikova, 2010). In addition, absence epilepsy is the petit-mal onset and never accompanied by aura and convulsions. In contrast to the absence epilepsy, the generalized tonic-clonic seizures are the grand-mal type of epilepsy (Wang et al., 2011). In clinic, the onset of this typical tonic-clonic seizures is characterized by the tense of muscles (Quiroga et al., 1997). In EEG, it can be identified as the paroxysmal fast activity of tonic phase with high frequency (>13 Hz) and low amplitude, which particularly accompanied by the evolutions into clonic phase with low frequency (<10 Hz) and high amplitude slow-wave oscillations (Ji et al., 2014). It has been demonstrated that both the absence and tonic-clonic seizures can generally appear in one complete recording from the same patient (Fong et al., 1998; Wakamoto et al., 2011; Shinnar et al., 2015). In particular, as seen from Figure 1, clinical and eletrographical evidences showed that absence seizures can evolve into the tonic-clonic oscillations of epilepsy (i.e., absence-tonic-clonic seizures, Figure 1B) (Mayville et al., 2000) and a tonic seizure can also be followed by the absence seizure (tonic-absence seizures, Figure 1A) (Shih and Hirsch, 2003). Although extensive observations and research have elucidated the mechanisms underlying the absence and tonic-clonic seizures, how these two seizures transit from each other remains an unanswered question in epileptology.

Neural field model can be used to characterize the macroscopic dynamics of neuronal populations. By introducing the disinhibition mechanism into the cerebral cortex, Fan et al. (2015) proposed a modified spatially-extended neural field model of cortex to computationally represent the physiologically plausible two-way transitions of epilepsy between absence and tonic-clonic seizures. However, the simultaneous recordings from cortex and thalamus of either the rodent animal models (Crunelli and Leresche, 2009) or clinical patients with epilepsies (Freestone et al., 2009) have shown that the pathological rhythms in cortex during epileptic seizures arise from the abnormal interactions within corticothalamic circuit (Coenen and Van Luijtelaar, 2003; Timofeev and Steriade, 2004; Millard et al., 2011; Sherman, 2012; Fan et al., 2016). This can also be supported by the interactions of ionic conductances in cells of both thalamus and cortex based on the specific anatomy of thalamocortical circuitry, which gives rise to the multiple modes of activities characterizing behavioral states of generalized epilepsy (Kush et al., 2016). These results provide the causal support for the involvement of subcortical thalamus in the initiations and developments of generalized epilepsies such as absence seizures and tonic-clonic seizures. Nevertheless, the thalamocortical mechanisms underlying the transitions between absence seizures and tonic-clonic seizures still need to be explored.

**Figure 2:**
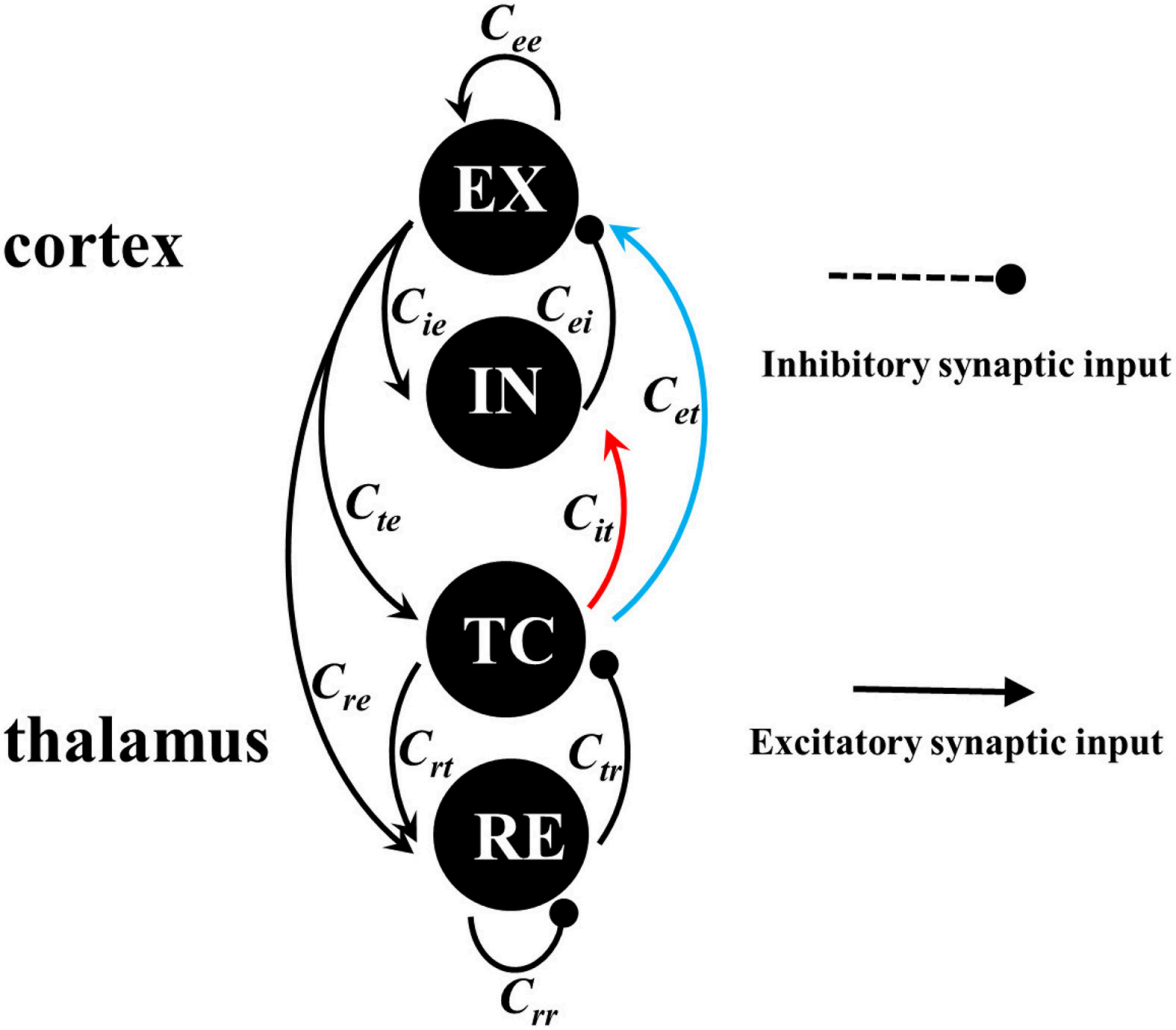
(Color online) Framework of the cortico-thalamic network. The cortical subsystem consists of excitatory (EX) pyramidal neuronal population and inhibitory (IN) interneuronal population. The thalamic subsystem consists of thalamus reticular (RE) nucleus and specific relay nuclei (TC). Excitatory projections are mediated by glutamate, which are denoted by the lines with arrow heads, while the inhibitory projections are mediated by *GABA* receptors, which are represented by the lines with round heads, respectively. The red and blue lines represent the feedforward inhibitory and excitatory connectivities, which are the only parameters we allow to vary.

In this paper, we employ the recently developed thalamocortical neural field model (Taylor et al., 2014) to characterize the macroscopic dynamics of neuronal populations within the thalamocortical circuit. Neural field models (Taylor et al., 2014; Wang and Wang, 2017) have been successfully applied to investigate the effect of stimulation on the SWD of absence seizures in epilepsy. As seen in Figure 2, the thalamocortical loop consists of cortical subsystem and thalamic subsystem. The cortical subsystem is composed of the excitatory (EX) neuronal population and inhibitory (IN) neuronal population, and the thalamic subsystem is composed of the thalamocortical (TC) relay neuronal populations and the thalamic reticular (RE) neuronal populations. The linking rules of intra-cortical EX-IN circuit and intra-thalamic TC-EX circuit, as well as the inter-thalamocortical loop, follow the work of Pinault and O'Brien (2005). However, in the previous model network, they didn't consider the feedforward inhibition from TC to IN. In fact, TC projects excitatory synaptic functions into both the cortical IN (inhibitory) and EX (excitatory) neuronal populations to comprehensively regulate the cortical firing activities by activating IN and EX, respectively. Therein, we refer to the projection from TC to IN as the feedforward inhibition of TC, which can first activate IN and the activated IN then further inhibits the abnormal discharges of EX. Hence, the feedforward inhibition, in effect, represents the TC-IN-EX pathway, in which the IN-EX pathway is inhibitory. Similarly, we refer to the direct projection from TC to EX as the feedforward excitation of TC. It is thought that in some cortical areas feedforward inhibition has even more stronger functions on cortex than the feedforward excitation from TC to EX (Porter et al., 2001; Sasaki et al., 2006; Cruikshank et al., 2007; Delevich et al., 2015; Herrera et al., 2015; Paz and Huguenard, 2015). Inspired by this, we propose a modified model by adding feedforward inhibition into the thalamocortical loop. Based on this modified model, we will systematically investigate the combined effects of feedforward inhibition and excitation from thalamus to cortex on the transition behaviors of epileptic seizures including tonic-clonic and absence seizures.

The organization of this paper is as follows. In Section 2, we review the model equations and the parametric variables of principal interest. The dynamical analysis for the model used here is also conducted in this section. In Section 3, we consider the feedforward effects from thalamus to cortex on the generalized epileptic seizures. In Sections 3.1 and 3.2, we compute the 1-D bifurcation diagrams to explore the effects of feedforward excitation and inhibition on the transition dynamics of generalized absence and tonic-clonic seizures, respectively. In Section 3.3, the combined effects of feedforward excitation and inhibition on the transition dynamics are explored by plotting 2-D bifurcation diagrams. In Section 4 we discuss these results.

## 2. Models and methods

### 2.1. Description of the model

In this paper, we propose a modified thalamocortical neural field model originally from Taylor et al. (2014), where we introduce the feedforward inhibition from TC to IN into the thalamocortical network. As seen in Figure 2, connection schematic of this modified neural field model is composed of the thalamocortical loop, which consists of the cortical EX-IN circuit and thalamic TC-RE circuit. The current improved model equations can be written as follows:

(1) $$\begin{array}{l} \frac{dEX}{dt}=\left(h_{e}-EX+C_{ee}f\left[EX\right]-C_{ei}f\left[IN\right]+C_{et}f\left[TC\right]\right)\tau_{e}, \end{array}$$

(2) $$\begin{array}{l} \frac{dIN}{dt}=\left(h_{i}-IN+C_{ie}f\left[EX\right]+C_{it}f\left[TC\right]\right)\tau_{i}, \end{array}$$

(3) $$\begin{array}{l} \frac{dTC}{dt}=\left(h_{t}-TC+C_{te}f\left[EX\right]-C_{tr}g\left[RE\right]\right)\tau_{t}, \end{array}$$

(4) $$\begin{array}{l} \frac{dRE}{dt}=\left(h_{r}-RE+C_{re}f\left[EX\right]+C_{rt}g\left[TC\right]-C_{rr}g\left[RE\right]\right)\tau_{r} \end{array}$$

Each equation is a rate equation with the units of voltage (activity) per time, where EX, IN, TC, RE are firing rates, i.e., the fractional firing activity in each neuronal populations, the parameters *h*_*e,i,t,r*_ are additive input constants to EX, IN, TC and RE, respectively. τ_*e,i,t,r*_ are the timescale parameters having the unit of *s*^−1^. *C*_*ee,ei,et,ie,it,te,tr,re,rt,rr*_ are the connectivity strengths within different neuronal populations whose linking rules essentially follow the experimentally known connection values. In particular, *C*_*it*_ and *C*_*et*_ represent the feedforward inhibition and excitation from TC to cortex. *f*[·] and *g*[·] are the activation functions of the cortical subsystem and thalamic subsystem, respectively:

(5) $$\begin{array}{l} f\left(x\right)=1/\left(1+\theta^{-x}\right) \end{array}$$

(6) $$\begin{array}{l} g\left(y\right)=\alpha y+\beta \end{array}$$

with *x* = EX, IN, TC and RE, *y* = TC, RE, and θ determines the steepness.

### 2.2. Dynamical analysis

The thalamocortical model can be represented as follows (Rodrigues et al., 2007, 2009):

(7) $$\begin{array}{l} \dot{X}=F\left(X\left(t\right),\nu \right), \end{array}$$

where F = (*F*_1_, *F*_2_, *F*_3_, *F*_4_)∈C^k^(ℝ^4^ × ℝ^21^, ℝ^4^) is the vector field for large enough *k*, X = (EX, IN, TC, RE), ν∈ℝ^21^ is the total parameter space, but in this present study, the parameters of interest are the feedforward excitation and inhibition of cortico-thalamic synaptic circuit, *w* = (*C*_*et*_, *C*_*it*_) ⊂ ν, due to its physiological relevance in studies of absence seizures. Because *C*_*et*_, *C*_*it*_ are the only parameters we allow to vary, in fact, F ∈*C*^*k*^(ℝ^4^ × ℝ^2^, ℝ^4^) and the system can be rewritten as

(8) $$\dot{X}=F\left(X\left(t\right)w\right)=\{\begin{array}{r} F_{1}\left(X\left(t\right),C_{et}\right)\\ F_{2}\left(X\left(t\right),C_{it}\right)\\ F_{3}\left(X\left(t\right)\right)\\ F_{4}\left(X\left(t\right)\right) \end{array}$$

Let X^*^(*t*) = (EX^*^, IN^*^, TC^*^, RE^*^) be fixed point of the system (8), i.e., equilibrium state being determined by setting the RHS (Borisyuk and Kirillov, 1992) of vector field of system (8) equal to 0,

(9) F(X(t)w)=0

The stability of equilibrium points can be analyzed by ensuring that the linearized version of (8) satisfies the Hartman-Groβman theorem (Kuznetsov et al., 1998). Consequently, we consider the Jacobian matrix (linearization matrix) of (8):

(10) $$\begin{array}{l} \text{J}=\left[\begin{array}{cccc} -\tau_{e}+\tau_{e}C_{ee}f^{\prime }\left(EX\right) & -\tau_{e}C_{ei}f^{\prime }\left(IN\right) & \tau_{e}C_{et}f^{\prime }\left(TC\right) & 0\\ \tau_{i}C_{ie}f^{\prime }\left(EX\right) & -\tau_{i} & \tau_{i}C_{it}f^{\prime }\left(TC\right) & 0\\ \tau_{t}C_{te}f^{\prime }\left(EX\right) & 0 & -\tau_{t} & \tau_{t}C_{tr}\alpha \\ \tau_{r}C_{re}f^{\prime }\left(EX\right) & 0 & \tau_{r}C_{rt}\alpha & -\tau_{r}-\tau_{r}C_{rr}\alpha \end{array}\right] \end{array}$$

Assume that for $C_{et}=C_{et}^{*}$ and $C_{it}=C_{it}^{*}$ at least one of the eigenvalues of the Jacobian matrix calculated at X^*^(*t*) is zero. This is valid if and only if the matrix determinant $\Delta \left(X^{*}\left(t\right),w^{*}\right)=|J{|}_{X^{*}\left(t\right),C_{et}^{*},C_{it}^{*}}=0$. We then consider the following equations

(11) $$\{\begin{array}{r} F(X(t),w)=0\\ \Delta_{\text{J}}(\text{X}(\text{t}),{\text{C}}_{\text{et}},{\text{C}}_{\text{it}})=0 \end{array}$$

which defines a curve in the six-dimensional space (EX, IN, TC, RE, C_et_, C_it_). The projection of this curve onto the plane w = (*C*_*et*_, *C*_*it*_) is called the fold point bifurcation curve.

In the 4-dimensional system of differential equations, the necessary and sufficient condition for a sum of two eigenvalues to be zero is that 3th Gurvitz determinant Δ_*G*_(*X*(*t*), *C*_*et*_, *C*_*it*_) = 0 (Gantmakher, 1967). If there exists a pair of eigenvalues of $J_{X^{*}\left(t\right)}$, λ_1_ and λ_2_, the sum of which is zero (i.e., either λ_1, 2_ = ±*iω*, or λ_1_= −λ_2_ are real), we consider the following equations,

(12) $$\{\begin{array}{r} F(X(t),w)=0\\ \Delta_{\text{G}}(\text{X}(\text{t}),{\text{C}}_{\text{et}},{\text{C}}_{\text{it}})=0 \end{array}$$

which define a curve in the six-dimension space (EX, IN, TC, RE, C_et_, C_it_). We call the projection of the curve onto the plane w = (*C*_*et*_, *C*_*it*_) the Andronov-Hopf bifurcation curve.

The double limit cycle curve can be defined by considering a fixed point of the Poincare map. Here the condition that the point (EX, IN, TC, RE, C_et_, C_it_) belongs to the double limit cycle curve can be written as a system of five equations, where four equations define that the point is the fixed point of Poincare map, and the fifth one defines that the point is a multiple fixed point of this map. The projection of this curve onto the plane w = (*C*_*et*_, *C*_*it*_) is called the double limit cycle curve. When parameters pass through this curve two limit cycles appear or disappear.

In addition, setting the linear change of variables to $\hat{X}=X-X^{*}$ and substituting appropriately into equations we obtain the following $\dot{\hat{X}}=F\left(\hat{X},w\right)$ where now F(0,w) = 0. Therefore, without loss of generalization, the state X = 0 is chosen to be the state of low level background activity. That is X = 0 is the steady state solutions of (1–4). *h*_*e,i,t,r*_ are external inputs into EX, IN, TC and RE neuronal populations, which can qualitatively induce the transitions between pathological state and non-seizure state of system. In the presence of external input (i.e., *h*_*e,i,t,r*_ ≠ 0), substituting X = 0 into the RHS of (1–4), we can quantitatively identify *h*_*e,i,t,r*_ as follows (Equation 13) through the interactive intensities among different neuronal populations, *C*_*ee,ei,et,ie,it,te,tr,re,rt,rr*_, within the thalamocortical circuit,

(13) $$\{\begin{array}{rr} 2h_{e}= & C_{ei}-C_{ee}-C_{et}\\ 2h_{i}= & -C_{ie}-C_{it}\\ 2h_{t}= & C_{tr}-C_{te}\\ 2h_{r}= & C_{rr}-C_{rt}-C_{re} \end{array}$$

Finally, epileptic absence seizures are characterized by SWD which is essentially the bursting oscillations. Bursting is one of the most important firing activities of neuronal systems. Rinzel (1985) firstly made some theoretical analysis on bursting and recognized that bursting exhibits a transition between a slow wave (or resting state) and a spiking state, owning to slow variation process modulating fast firing activities. In this system, TC can be considered as the slow variables in the model subsystem (3), which controls the dynamics of the fast subsystem (1), (2), and (4). Therefore, the fast-slow dynamics analysis plays an important role for the dynamical behavior of firing patterns (Rinzel, 1985; Izhikevich, 2000; Duan et al., 2008), especially bursting-like SWD discharges of absence seizure in epilepsy.

### 2.3. Simulation method and data analysis

All model parameters used to make simulations in this manuscript are listed in Table 1. The standard fourth-order Runge-Kutta integration scheme under the MATLAB (MathWorks, USA) simulating environment was used with Equations (1–4). The model EEG is taken as the mean of two cortical populations, EX and IN. Both the bifurcation and frequency analysis (Chen et al., 2014, 2015, 2017) are utilized to characterize the critical state transitions and neural oscillations generated by our model. Firstly, the bifurcation analysis is performed by calculating the stable local minimum and maximum values of the mean for EX and IN. Furthermore, to evaluate the dominant frequency of neural oscillations, the power spectral density (PSD) is estimated using the fast Fourier transform (FFT) for the time series of the mean for EX and IN. Then the maximum peak frequency is defined as the dominant frequency of neural oscillations. We further investigated the interesting dynamical features of the modified model numerically using the software package XPPAut (Ermentrout, 2002). The bifurcation diagrams were computed by using AUTO as incorporated in XPPAut.

**Table 1:** Parameter values of system (1)–(4) used in the manuscript.

**Symbol**	**Interpretation**	**Value**
*C*_*ee*_	EX → EX connectivity strength	1.8
*C*_*ie*_	EX → IN connectivity strength	4
*C*_*te*_	EX → TC connectivity strength	3
*C*_*re*_	EX → RE connectivity strength	3
*C*_*rt*_	TC → RE connectivity strength	10.5
*C*_*rr*_	RE → RE connectivity strength	0.2
*C*_*tr*_	RE → TC connectivity strength	0.2
*C*_*it*_	TC → IN connectivity strength	varied
*C*_*et*_	TC → EX connectivity strength	varied
*C*_*ei*_	IN → EX connectivity strength	1.8
τ_*e*_	EX timescale	26 s^−1^
τ_*i*_	IN timescale	32.5 s^−1^
τ_*t*_	TC timescale	2.6 s^−1^
τ_*r*_	RE timescale	2.6 s^−1^
*h*_*e*_	Input EX	−0.35
*h*_*i*_	Input IN	−3.4
*h*_*t*_	Input TC	−2
*h*_*r*_	Input RE	−5
θ	Sigmoid steepness	2.5 × 10^5^
α	Linear intersection steepness	2.8
β	Linear intersection offset	0.5

*EX, excitatory pyramidal neuron; IN, inhibitory interneurons; TC, thalamocortical relay cells; RE, thalamic reticular nucleus*.

## 3. Results

### 3.1. Feedforward excitation TC → EX inducing tonic-absence seizures with weak feedforward inhibition

Tonic-absence seizures, originally termed by Shih and Hirsch (2003), consist of a tonic seizure with generalized paroxysmal fast activity (>13 Hz) which is followed by an absence seizure with generalized slow spike-wave activity (~2–4 Hz). Clinically and electrographically, this is a poorly characterized seizure type in patients with symptomatic generalized epilepsy. The typical tonic-absence EEG pattern can be found in the Figure 1A, modified from Figure 2A in the work of Shih and Hirsch (2003). In this section, we computationally investigate the effect of feedforward excitation from TC to EX, i.e., *C*_*et*_, on the generation of tonic-absence seizures, based on the modified thalamocortical network model.

#### 3.1.1. Firing transitions of modified model system

**Figure 3:**
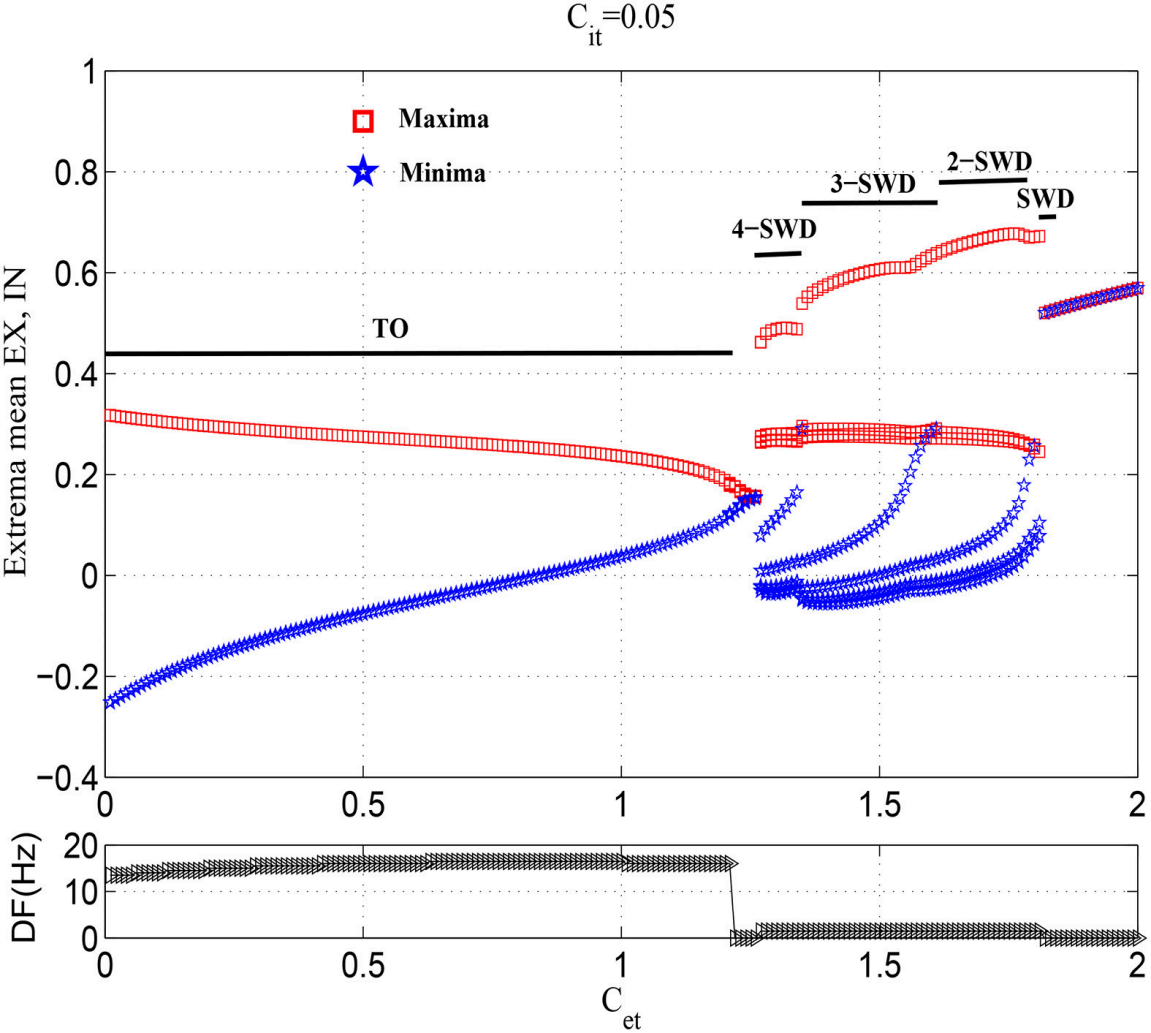
Bifurcation diagrams (**upper panel**) and corresponding dominant frequencies (**lower panel**) showing model dynamics of a single compartment over changes in *C*_*et*_ with *C*_*it*_ fixed at 0.05. The system transits from the tonic oscillations (TO) to 4-spike and wave discharges (4-SWD), 3-spike and wave discharges (3-SWD), 2-spike and wave discharges (2-SWD), spike and wave discharges (SWD) and to high saturated state (HS), respectively.

**Figure 4:**
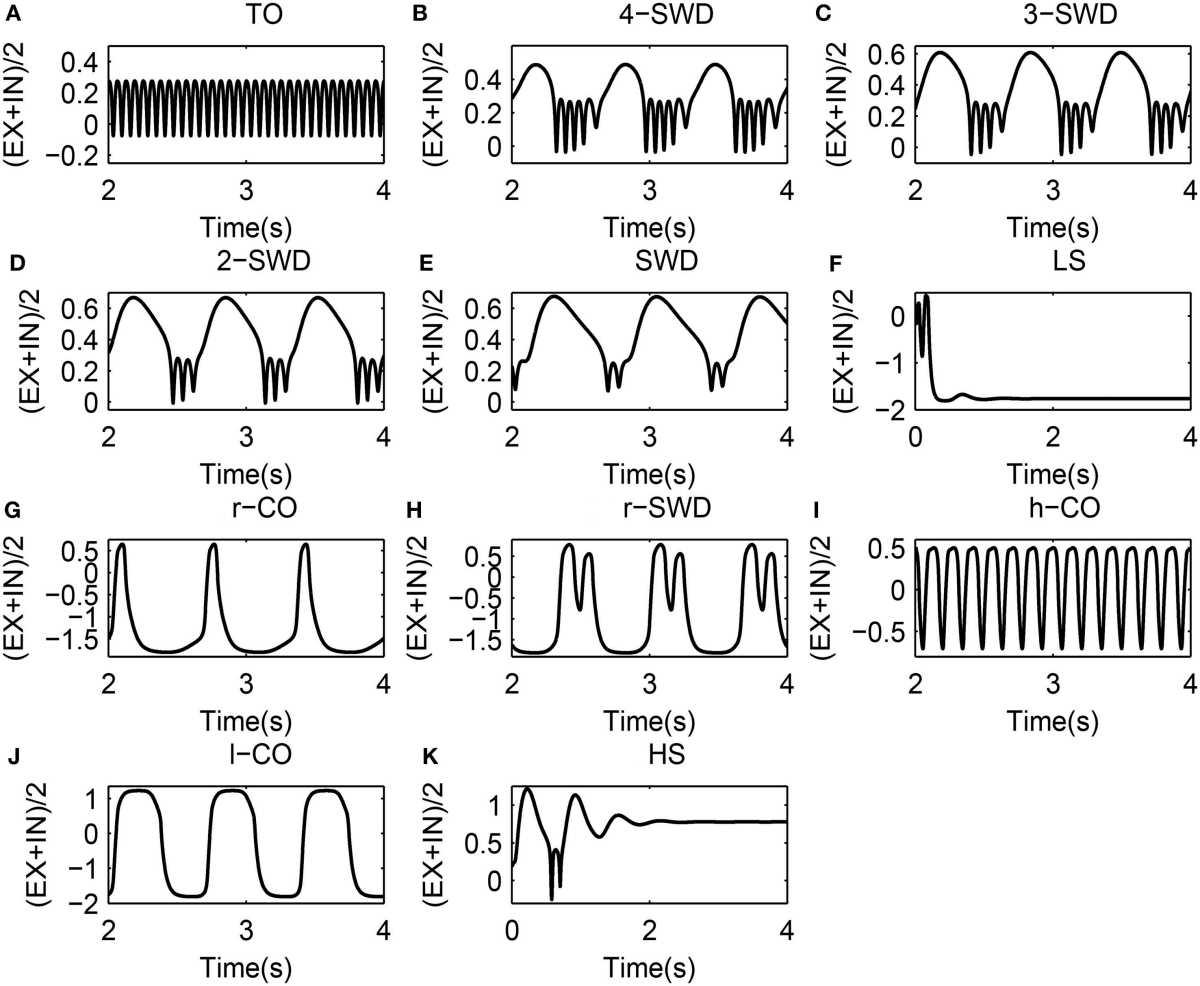
11 solutions along with the marker style used to represent them. **(A)** TO (tonic oscillations): *C*_*it*_ = 0.05, *C*_*et*_ = 0.5; **(B)** 4-SWD (4-spike and wave diacharges): *C*_*it*_ = 0.05, *C*_*et*_ = 1.3; **(C)** 3-SWD (3-spike and wave discharges): *C*_*it*_ = 0.05, *C*_*et*_ = 1.5; **(D)** 2-SWD (2-spike and wave discharges): *C*_*it*_ = 0.05, *C*_*et*_ = 1.7; **(E)** SWD (spike and wave discharges): *C*_*it*_ = 0.05, *C*_*et*_ = 1.81; **(F)** LS (low saturated state): *C*_*it*_ = 1.0, *C*_*et*_=0.05; **(G)**r-CO (reversed clonic oscillations): *C*_*it*_ = 1.0, *C*_*et*_ = 0.2; **(H)** r-SWD (reversed SWD): *C*_*it*_ = 1.0, *C*_*et*_ = 0.4; **(I)** h-CO (high-frequency CO): *C*_*it*_ = 1.0, *C*_*et*_ = 0.8; **(J)** l-CO (low-frequency CO): *C*_*it*_ = 1.0, *C*_*et*_ = 1.2; **(K)** HS (High saturated state): *C*_*it*_ = 1.0, *C*_*et*_ = 2. For clarity, these solutions are also indicated by text labels in each figure.

Figure 3 gives an overall view of cortical various state transitions with the increasing of *C*_*et*_ and fixing *C*_*it*_ = 0.05, i.e., under the condition of weak feedforward inhibition from TC to IN. It is seen from the upper panel of Figure 3 that the small values of *C*_*et*_, e.g., *C*_*et*_ <≈1.2, can induce the simple oscillations. The exemplary time series for this is given in Figure 4A. Compared to the lower panel of Figure 3, it is seen that the corresponding dominant frequencies of simple oscillations are larger than 13 Hz, which hence represents the generalized tonic seizures of epilepsy. Electrophysiologically, this is because that the weak feedforward inhibition is unable to effectively activate the cortical inhibitory neuronal populations (IN), which cannot further confront the abnormal discharges of excitatory neuronal populations (EX). Especially, it can also be observed that with the increasing of *C*_*et*_, the amplitudes of tonic oscillations gradually decrease, while the vibration frequencies are amplified step by step. As 1.22 <≈ *C*_*et*_ <≈1.25, tonic oscillations decay to steady values. Accordingly, the system enters into the transient low saturated (LS) firing states.

However, as the further increasing of *C*_*et*_, the system transits into the multi-spike and wave discharges (m-SWD) from the low saturated firing states. Firstly, the system shows the periodic 4-spike and wave discharges (4-SWD) within the parameter region 1.25 <≈ *C*_*et*_ <≈1.35. Then, the system successively transits from 4-SWD to the periodic 3-/2-spike and wave discharges (3-/2-SWD), corresponding to the parameter regions, 1.35 <≈ *C*_*et*_ <≈1.6 and 1.6 <≈ *C*_*et*_ <≈1.78, respectively. Immediately after the m-SWD, the system transits into the SWD (i.e., periodic 1-spike and wave discharges, SWD), corresponding to 1.78 <≈ *C*_*et*_ <≈1.8, which is the characteristic of epileptic absence seizures. Finally, for the much large values of *C*_*et*_, the system shows the high saturated (HS) firing states. Overall, the feedforward inhibition, i.e., *C*_*et*_, can induce the transitions from tonic oscillations to SWD discharges, which mathematically describes the clinically and electrographically observed tonic-absence seizures process of epilepsy.

The exemplary time series for the solutions of 4-/3-/2-/1-SWD are displayed in the Figures 4B–E, which also serve as the legends for the subsequent bifurcation diagrams. In order to make a link between the above-mentioned time series and the real patient EEG data, Figure 1C gives the EEG traces from seizure database, taken from two different patients with childhood absence epilepsy (CAE, upper and middle panels) and one patient with juvenile absence epilepsy (JAE, lower panel). Specifically, EEG traces show the 1-SWD and 2-SWD discharges for patients with CAE and 3-SWD discharges for patient with JAE, respectivley. In particular, through the qualitative comparison between the human EEG data (Figure 1C) and the time traces of cortico-thalamic model (Figures 4C–E) we can find an interesting qualitative agreement between the data and the model.

#### 3.1.2. The dynamical mechanisms underlying the tonic-absence seizures

**Figure 5:**
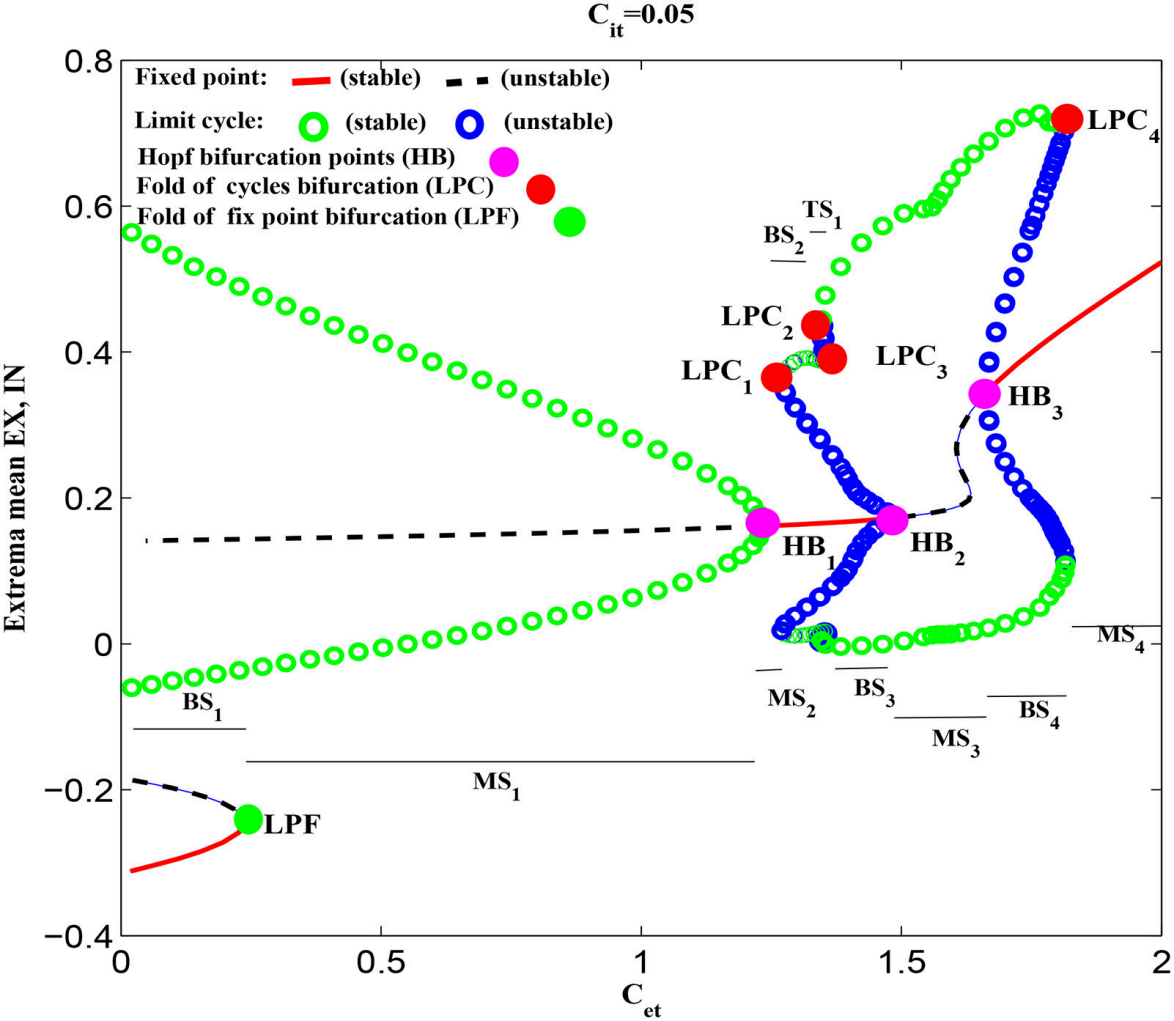
(Color online) Dynamical bifurcation diagram corresponding to the state transitions in Figure 3. As *C*_*et*_ increasing, the system successively undergoes the fold of fixed point bifurcation (LPF), Hopf bifurcation (*HB*_1_), the fold of cycles bifurcation (*LPC*_1_), *LPC*_2_, *LPC*_3_, *HB*_2_, *HB*_3_, and *LPC*_4_, respectively. The parameter regions consist of monostable region (“MS”) including stable fixed point (*MS*_2_, *MS*_4_), and stable limit cycles (*MS*_1_, *MS*_3_), bistable regions (“BS,” *BS*_1_ − *BS*_4_) composed of stable limit cycle and stable fixed point, and tristable region (“TS”) composed of stable fixed point and two stable limit cycles, respectively.

Figure 5 shows the dynamical explanations for the bifurcation diagram of these state behaviors corresponding to Figure 3 and Figure 4. It is seen that for small values of *C*_*et*_, the solutions of the system are either fixed points or tonic oscillation (TO). Among these solutions, there is a region of bistability (*BS*_1_) consisting of a stable fixed point and a stable limit cycle, corresponding to *C*_*et*_ <≈0.24. At *C*_*et*_ ≈0.24, the stable fixed point disappears due to a fold of fixed point bifurcation (LPF). After that, the system enters into the monostable regions composed of stable limit cycle and stable focus, corresponding to 0.24 <≈ *C*_*et*_ <≈1.22 (i.e., *MS*_1_) and 1.22 <≈ *C*_*et*_ <≈1.25 (i.e., *MS*_2_), respectively. In particular, at *C*_*et*_ = 1.22, the stabilities of focuses switch following a supercritical Hopf bifurcation (*HB*_1_). The m-SWD solutions emerge for larger *C*_*et*_ (e.g., 1.25 <≈ *C*_*et*_ <≈1.8) successively following a fold of cycles bifurcation (*LPC*_1_), *LPC*_2_, *LPC*_3_, *HB*_2_ and *HB*_3_ bifurcations, which give rise to the monostable regions (*MS*_3_), bistable regions (*BS*_2_, *BS*_3_, *BS*_4_) and tristable region (*TS*_1_), respectively. In particular, compared to the Figure 3, SWD solution occurs in the bistable region of 1.78 <≈ *C*_*et*_ <≈1.8. Finally, the system undergoes another *LPC*_4_ and enters into the monostable fixed points (*MS*_4_).

It is noted that the system status in the multistable regions is dependent on the initial values of the four state variables. From the mathematical standpoint, there exist separating manifolds, i.e., separatrix, between the attraction basins of various stable states. Specifically, when the initial values of the four state variables are close to the side of separatrix near the stable fixed points, the system will converge to the steady states, otherwise the system will show the tonic oscillations or m-SWD discharges when the initial values fall into the attraction basin of stable limit cycle. In particular, during the simulations, all the initial values of the four state variables are set to [0.1724 0.1787 – 0.0818 0.2775] which is within the attraction basins of stable limit cycle. Hence the system displays the periodic simple tonic oscillations or m-SWD discharges. Note that, the dynamical explanations also serve as a legend for the subsequent state bifurcation diagrams.

#### 3.1.3. Fast-slow decomposition analysis

**Figure 6:**
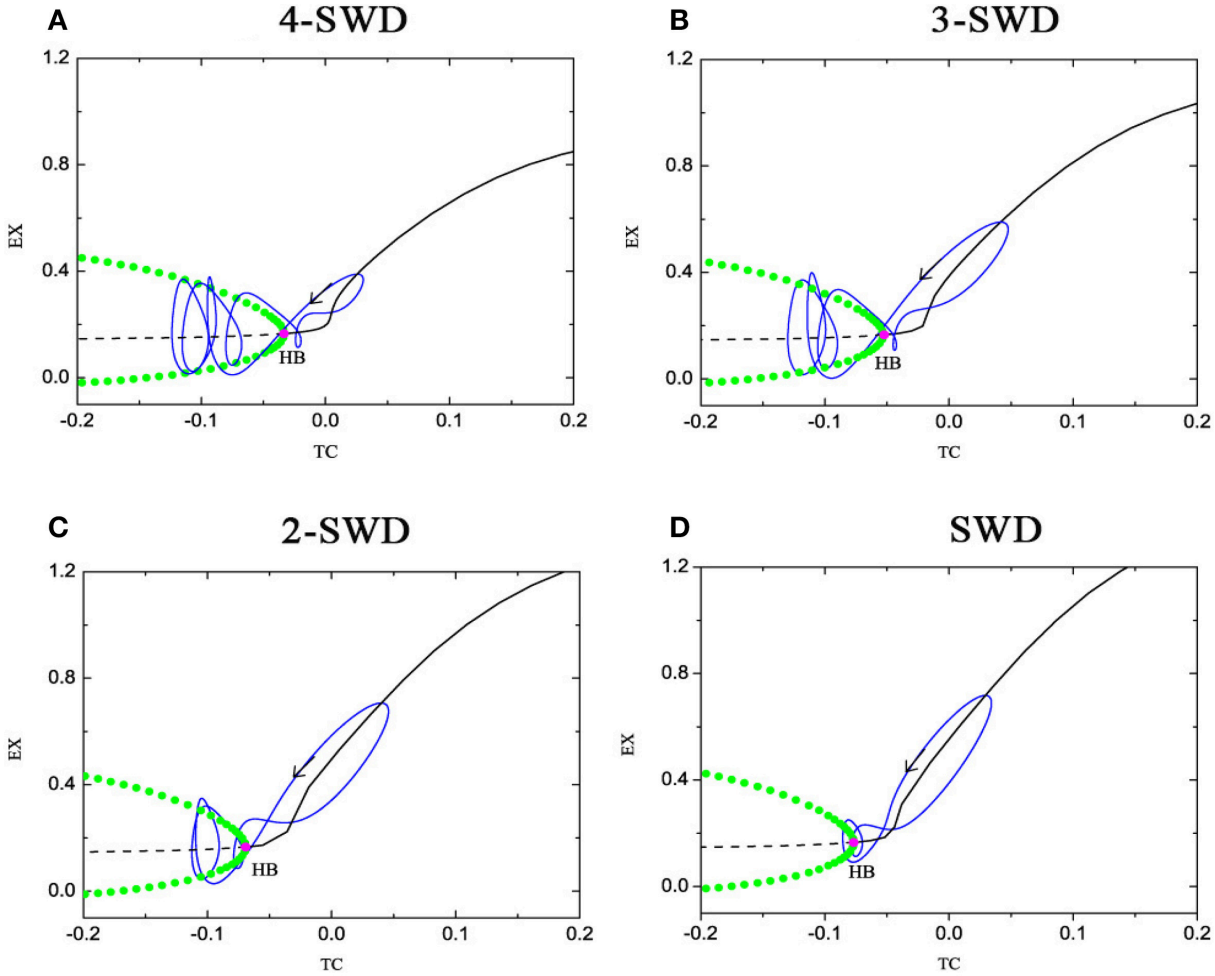
(Color online) Fast-slow dynamical analysis of fast subsystem (1), (2), and (4) with respect to the slow variable TC at **(A)** *C*_*it*_ = 0.05, *C*_*et*_ = 1.3; **(B)** *C*_*it*_ = 0, 05, *C*_*et*_ = 1.5; **(C)** *C*_*it*_ = 0.05, *C*_*et*_ = 1.7; **(D)** *C*_*it*_ = 0.05, *C*_*et*_ = 1.81, corresponding to Figures 4B–E, respectively. The solid and dashed curve represent the stable and unstable focus. HB refer to the supercritical Hopf bifurcation and the trajectory of the system (1)–(4) is also superimposed on it. The other parameter values are same as that in Figure 5.

The fast-slow dynamics underlies the basis of dynamical behavior of bursting firing patterns (Rinzel, 1985; Izhikevich, 2000; Duan et al., 2008), especially bursting-like SWD discharges of absence seizure in epilepsy. Figure 6 shows the fast-slow dynamical analysis for the multi-spike and wave discharges (m-SWD). The equilibrium points form a lay down “L”-shaped curve, in which the stable and unstable focus curves are described with the solid and dashed line respectively, which are separated by the supercritical Hopf bifurcation point (HB). The stable limit cycles occur at the supercritical Hopf bifurcation (HB). The maximal and minimal values of the limit cycles of EX are described with green solid dot. The oscillations (spikes) emerge because of the stable limit cycles which bifurcate from the HB point. For 4-spike and wave discharges at *C*_*et*_ = 1.3, the supercritical Hopf bifurcation occurs at TC = −0.03338 (Figure 6A), 3-spike at TC = −0.0532311 (Figure 6B), 2-spike at TC = −0.06917 (Figure 6C) and 1-spike at TC = −0.07676 (Figure 6D). With the increase of parameter *C*_*et*_, the HB moves left which results in the decrease of number and amplitude of spikes. So the bursting transits from 4-spike to 1-spike.

### 3.2. Feedforward inhibition TC → IN inducing absence-clonic seizures with strong feedforward excitation

**Figure 7:**
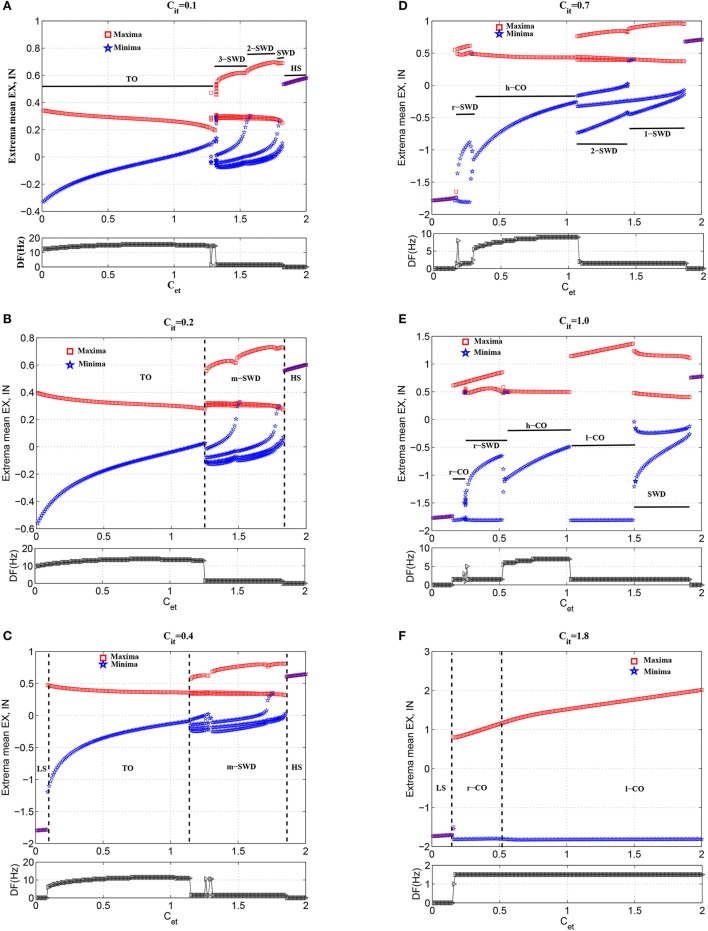
Bifurcation diagrams (upper panels) and corresponding dominant frequencies (lower panels) showing model dynamics of a single compartment over changes in Cet with Cit fixed at **(A)** 0.1, **(B)** 0.2, **(C)** 0.4, **(D)** 0.7, **(E)** 1.0, and **(F)** 1.8, respectively. For clarity, TO, m-SWD (*m* = 1,2,3), r-SWD (reversed SWD discharges), LS (low saturated state), HS, and r/h/l-CO (reversed/high-frequency/low-frequency clonic oscillations) solutions are indicated by text labels in each figure.

In order to investigate the modulation functions of feedforward inhibition on the feedforward excitation inducing tonic-absence seizures, we gradually increase the feedforward inhibition, i.e., *C*_*it*_, to observe the state transitions from the various phases of tonic-absence seizures. Figure 7 captures the typical state transitions under the different functions of feedforward inhibitions with fixing *C*_*it*_ at 0.1 (Figure 7A), 0.2 (Figure 7B), 0.4 (Figure 7C), 0.7 (Figure 7D), 1.0 (Figure 7E), and 1.8 (Figure 7F), respectively. In what follows, for the sake of clearness, the investigations for the state transitions are mainly focused on the three parameters intervals of *C*_*et*_, i.e., *C*_*et*_ ∈ [0, 0.2], *C*_*et*_ ∈ [0.2, 0.6] and *C*_*et*_ ∈ [0.6, 2], respectively.

#### 3.2.1. Delayed onset of epileptiform discharges

As shown in Figures 7A,B, the weak feedforward inhibition, *C*_*it*_, can not effectively inhibit the cortical activity motivated by the feedforward excitations, *C*_*et*_, which can immediately induce the high-frequency tonic oscillations. However, as shown in Figure 7C, with the feedforward inhibition getting large, e.g., *C*_*it*_ = 0.4, the feedforward excitation induced tonic oscillations can be suppressed and the system transits into the low saturated state (LS). This means feedforward inhibition can delay the onset of epileptic tonic seizures by inhibiting the abnormal epileptic activities activated by feedforward excitations. Interestingly, compared Figure 7C with Figure 7D, we can find that with *C*_*et*_ further increasing the postponed onset of tonic oscillations in the modified model can be prolonged. Dynamically, this state transition from tonic oscillations to low saturated firing is attributed to the bistability mechanism composed of stable limit cycle and stable fixed point. Specifically, when *C*_*it*_ is set at small values, e.g., *C*_*it*_ = 0.1 or 0.2, weak feedforward inhibition fails to drive the system to escape from the attractor basin of limit cycle representing the tonic oscillations. However, as *C*_*it*_ increased, e.g., *C*_*it*_ = 0.4, for the small feedforward excitation, e.g., *C*_*et*_ < 0.1, which leads to the tonic oscillations with lower dominant frequency, the feedforward inhibition can successfully drive the system beyond the separatrix and into the attractor basin of fixed point, and the system will eventually converge to the steady saturated states. In particular, with *C*_*it*_ further increasing, the tonic oscillations with higher-frequency induced by the larger 0.1 < *C*_*et*_ < 0.2 can also be terminated through driving the system to escape away from the attractor basin of limit cycle with smaller period. Exemplary time series for the solutions of low saturated firing and tonic oscillations are shown in Figures 4A,F.

#### 3.2.2. Seizure transitions between m-SWD (r-SWD) discharges and clonic (r-Clonic) oscillations

Compared Figures 7D–F to Figure 7C, it can be seen that for 0.2 < *C*_*et*_ < 0.6, the larger feedforward inhibitions can not abate the tonic oscillations again, but induce the more richer slow-wave dynamics. In particular, for *C*_*it*_ = 0.7 (Figure 7D), the whole system shows the low-frequency (<10 Hz) oscillations, along with the shift of tonic oscillations (TO) to high-frequency clonic oscillations (h-CO). Also, for 0.2 < *C*_*et*_ < 0.3 the reversed SWD discharges (r-SWD) emerge from the tonic-clonic oscillations. With the further increasing of *C*_*it*_, e.g., *C*_*it*_ = 1.0, r-SWD discharges are gradually transformed into the reversed clonic oscillations (r-CO). Along with this transformations is the emergence of new r-SWD discharges from the h-CO oscillations in the larger parameter region, 0.3 < *C*_*et*_ < 0.6. For the much large *C*_*it*_ = 1.8, r-SWD discharges can eventually transit into the r-CO oscillations. In some sense, under the condition of weak feedforward excitation (e.g., *C*_*et*_ < 0.6), with the increasing of feedforward inhibition, the state evolutions from tonic-clonic oscillations to reversed SWD (r-SWD) discharges and clonic oscillations (r-CO) describe the physiological transitions between tonic-clonic and absence epileptic seizures. Exemplary time series for the solutions of r-CO, r-SWD and h-CO are shown in Figures 4G,H,I.

In addition, Figures 7A–E also show that with *C*_*it*_ increasing from 0.1, 0.2, 0.4 to 0.7, the parameter region of *C*_*et*_ corresponding to SWD is getting larger. And within the parameter region of larger *C*_*et*_ corresponding to m-SWD, the system transits from 4-SWD, 3-SWD to 2-SWD, respectively. As *C*_*it*_ further increased, e.g., *C*_*it*_ = 1.0, the 2-SWD and SWD discharges of the system within 1.0 < *C*_*et*_ < 1.5 can be further suppressed and turned into low-frequency (<5 Hz) clonic oscillations (l-CO, see Figure 4J). For the much large *C*_*it*_ = 1.8, within 0.6 < *C*_*et*_ < 2.0, the whole system is inhibited by the large enough feedforward inhibitions and only shows the ~1.5 Hz slow-wave clonic oscillations. These transitions from m-SWD (*m* = 1,2,3,4) to clonic oscillations induced by the feedforward inhibition represent the physiologically observed absence-clonic seizures shown in Figure 1B.

### 3.3. Combined effects of feedforward inhibition and excitation on epileptic seizures

**Figure 8:**
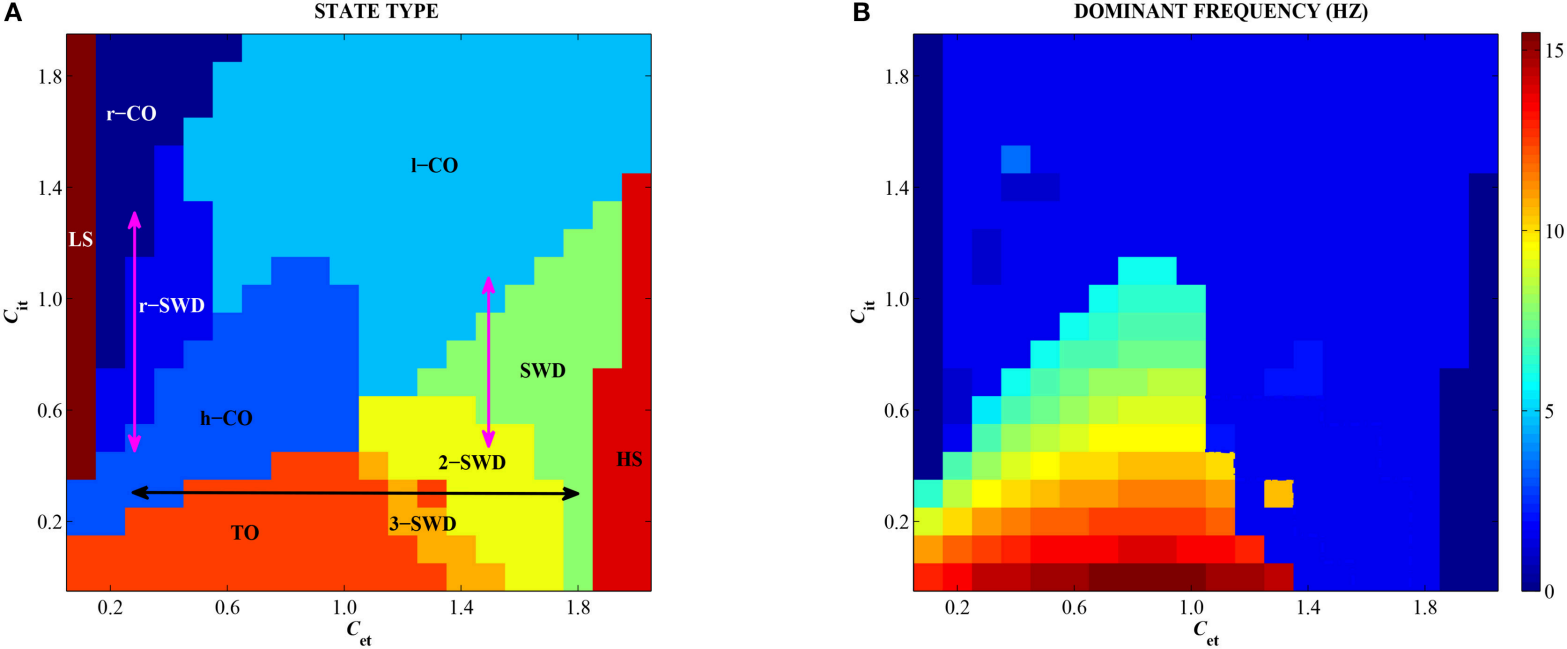
(Color online) Different firing states **(A)** and variations of corresponding dominant frequency **(B)** of the single modified Taylor model are shown on the parameter plane (*C*_*et*_, *C*_*it*_). TO, m/r-SWD (*m* = 1,2,3), LS, HS, and r/h/l-CO solutions are indicated by text labels. Double arrows indicate the possible transition pathways between different firing states.

In order to observe the overall dynamics transition of the modified Taylor model as shown in Figure 2, we depict the state transitions (Figure 8A) and corresponding dominant frequency (Figure 8B) as the feedforward excitation and inhibition from subcortical thalamus to cortex, *C*_*et*_ and *C*_*it*_, are changed in 2-D plane [0, 2] × [0, 2]. It can be seen in Figure 8A that there are ten types of firing states, which are denoted as: LS/HS: low/high saturated firings, TO: tonic oscillations with high frequency (>10 Hz) and low amplitude, h/l-CO: clonic oscillations with high (>5 Hz & <10 Hz) and low (~2 Hz) frequencies, r-CO: reversed clonic oscillations, m-SWD: multi-spike and wave discharges (*m* = 2,3), SWD: spike and wave discharges (*m* = 1), r-SWD: reversed SWD discharges.

Specifically, for the both small values of *C*_*et*_ and *C*_*it*_, the system exhibits the epileptic tonic-clonic oscillations. By contrast, the much large *C*_*et*_ and *C*_*it*_ can suppress the system to show low-frequency clonic oscillations. However, the unbalanced functions between feedforward excitation and inhibition can induce the epileptic spike-wave discharges. For example, for the small *C*_*it*_ (e.g., <1, i.e., the weak feedforward inhibition), the strong feedforward excitation (e.g., *C*_*et*_ > 1) can activate the system to display typical m-SWD (*m* = 2, 3) and SWD (*m* = 1) discharges of absence seizures in epilepsy. Conversely, for the small *C*_*et*_ (e.g., <0.6, i.e., the weak feedforward excitation), the stronger feedforward inhibition can induce the reversed SWD (r-SWD) discharges and clonic (r-CO) oscillations. In particular, the horizontal double black arrow and the left vertical double pink arrow represent the physiologically observed epileptic transitions from tonic-clonic oscillations to absence seizures (see Figure 1A), which corresponds to the results of Sections 3.1.1 and 3.2.2, respectively. The right vertical pink arrow represents the physiologically observed seizure transitions from absence seizures to clonic oscillations of epilepsy (see Figure 1B), which also corresponds to the result of Section 3.2.2. In addition, non-oscillation activities, i.e., saturated states, occur for the small *C*_*et*_ and large *C*_*it*_, or the large *C*_*et*_ and small *C*_*it*_, corresponding to regions LS and HS, respectively. What's more, as shown in Figure 8B, only both weak feedforward excitation and inhibition, corresponding the parameter region of small *C*_*et*_ and *C*_*it*_, can result in the high-frequency oscillations, while the other parameter regions correspond to the low-frequency oscillations.

## 4. Conclusion

In this paper, using a modified thalamocortical neural field model network, we systematically investigated the combined effects of feedforward inhibition and excitation from thalamus to cortex on the epileptic seizure transitions. Electrophysiological experiments have long revealed the existence of two-way transitions between absence and tonic-clonic epileptic seizures in the cerebral cortex (Mayville et al., 2000; Shih and Hirsch, 2003). By computational modeling, we first demonstrated that under the condition of weak feedforward inhibition, the enhancement of feedforward excitation can induce the transitions from tonic-clonic oscillations of epilepsy to SWD of absence seizures. More importantly, we showed that the phase of absence seizures corresponding to strong feedforward excitation can be further transformed into the clonic oscillations with the enhancement of feedforward inhibition, representing the epileptic absence-clonic seizure transitions. Our findings highlight the functional importance of thalamic feedforward inhibition and excitation, which might contribute to the emergences and transitions of epileptic seizures including tonic-clonic oscillations and absence epilepsy.

Inhibitory interneuronal population plays a crucial role in the seizure suppressions. It can confront the abnormal discharges of excitatory neuronal population to control epileptic seizures. Hence, our model can be extended to investigate the opposite issues such as the modulations or suppressions of absence seizures by balancing thalamic feedforward inhibition and excitation. However, it remains difficult to discern whether the feedforward inhibition are leading the feedforward excitation or vice versa. In addition, previous experimental study has shown that RE can facilitate sharing information and consolidate new memories by coordinating the slow-wave oscillations of different brain regions (Lewis et al., 2015). Hence, we will explore the modulating role of RE for the feedforward effect of TC on the transitional dynamics of epileptic seizures.

## Author contributions

DF, LD, QW, and GL designed and performed the research as well as wrote the paper.

### Conflict of interest statement

The authors declare that the research was conducted in the absence of any commercial or financial relationships that could be construed as a potential conflict of interest.
